# Spontaneous calcification process in primary renal cells from a medullary sponge kidney patient harbouring a GDNF mutation

**DOI:** 10.1111/jcmm.12514

**Published:** 2015-02-18

**Authors:** Federica Mezzabotta, Rosalba Cristofaro, Monica Ceol, Dorella Del Prete, Giovanna Priante, Alessandra Familiari, Antonia Fabris, Angela D'Angelo, Giovanni Gambaro, Franca Anglani

**Affiliations:** a Laboratory of Histomorphology and Molecular Biology of the Kidney, Nephrology Division, Department of Medicine DIMED, University of PaduaPadua, Italy; b Nephrology Division, Department of Biomedical and Surgical Sciences, University Hospital of VeronaVerona, Italy; c Nephrology Division, Department of Internal Medicine and Medical Specialties, Columbus-Gemelli University Hospital, Catholic UniversityRome, Italy

**Keywords:** nephrocalcinosis, medullary sponge kidney, GDNF, mesenchymal stromal stem cells

## Abstract

Medullary nephrocalcinosis is a hallmark of medullary sponge kidney (MSK). We had the opportunity to study a spontaneous calcification process *in vitro* by utilizing the renal cells of a patient with MSK who was heterozygous for the c.-27 + 18G>A variant in the GDNF gene encoding glial cell-derived neurotrophic factor. The cells were obtained by collagenase digestion of papillary tissues from the MSK patient and from two patients who had no MSK or nephrocalcinosis. These cells were typed by immunocytochemistry, and the presence of mineral deposits was studied using von Kossa staining, scanning electron microscopy analysis and an ALP assay. Osteoblastic lineage markers were studied using immunocytochemistry and RT-PCR. Staminality markers were also analysed using flow cytometry, magnetic cell separation technology, immunocytochemistry and RT-PCR. Starting from p2, MSK and control cells formed nodules with a behaviour similar to that of calcifying pericytes; however, Ca_2_PO_4_ was only found in the MSK cultures. The MSK cells had morphologies and immunophenotypes resembling those of pericytes or stromal stem cells and were positive for vimentin, ZO1, αSMA and CD146. In addition, the MSK cells expressed osteocalcin and osteonectin, indicating an osteoblast-like phenotype. In contrast to the control cells, GDNF was down-regulated in the MSK cells. Stable GDNF knockdown was established in the HK2 cell line and was found to promote Ca_2_PO_4_ deposition when the cells were incubated with calcifying medium by regulating the osteonectin/osteopontin ratio in favour of osteonectin. Our data indicate that the human papilla may be a perivascular niche in which pericyte/stromal-like cells can undergo osteogenic differentiation under particular conditions and suggest that GDNF down-regulation may have influenced the observed phenomenon.

## Introduction

Many *in vitro* and *in vivo* studies focusing on the mechanisms underlying calcium nephrolithiasis have provided evidence of a condition frequently associated with nephrocalcinosis, a microscopic renal crystal deposition that can occur within the tubular lumen (intratubular nephrocalcinosis) or in the interstitium (interstitial nephrocalcinosis) 1,2.

Medullary nephrocalcinosis is the typical pattern observed in 98% of cases of human nephrocalcinosis, with clusters of calcification occurring around each renal pyramid and is common in patients with metabolic conditions that predispose them to renal calcium stones, such as medullary sponge kidney (MSK).

Although the clinical, biochemical and genetic aspects of the diseases that cause nephrocalcinosis have been fairly thoroughly elucidated, little is known about the specific cellular events or the histopathology of such calcifications.

The most accredited hypothesis that explains the onset of interstitial nephrocalcinosis is purely physicochemical, relating to spontaneous Ca_2_PO_4_ crystallization in the interstitium because of oversaturation of Ca_2_PO_4_ salts in this milieu 3. The theory that nephrocalcinosis is a process driven by osteogenic cells was first proposed by our group 4. The notion that resident renal cells could be prompted to trans-differentiate or differentiate along an osteogenic lineage was based on the following observations: Miyazawa *et al*. 5 reported data demonstrating that CaOx crystals up-regulated vimentin in normal rat kidney proximal cells, Peerapen *et al*. 6 showed that CaOx crystals caused a marked decrease in tight junction proteins in Madin-Darby canine kidney cells, and Kumar *et al*. 7 found that rat inner medullary collecting duct cells grown in a calcifying medium formed calcifying nodules that were positive for typical bone proteins. It is currently unclear whether this phenomenon also occurs in human kidneys.

Medullary sponge kidney is a renal malformation typically associated with medullary nephrocalcinosis and renal stones. In previous studies, we advanced the hypothesis that MSK may occur because of a disruption of the metanephric mesenchyme–ureteric bud interface caused by mutations in the GDNF gene, which encodes glial cell-derived neurotrophic factor 8,9.

We had the unique opportunity to investigate the hypothesis that nephrocalcinosis originates from an osteogenic cell-driven process by studying the *in vitro* behaviour of renal cells obtained from a patient with MSK carrying a GDNF mutation. The process of spontaneous calcification was observed and investigated in these cells.

## Materials and methods

### MSK diagnosis

Medullary sponge kidney is commonly diagnosed during the work-up for recurrent calcium nephrolithiasis based on the strength of typical MSK images obtained *via* IV urography or, more recently, uro-CT scan after other causes of nephrocalcinosis are excluded. To be eligible for an MSK diagnosis, patients must present involvement of both kidneys with typical nephrocalcinosis and/or cystic features at the papillary level in at least two papillae per kidney. The demonstration of papillary precalyceal ectasia, which is documented on images obtained at least 10 min. after injection of the contrast medium, without compression manoeuvres and without signs of obstruction is mandatory for MSK diagnosis.

### Renal papillae from open surgery

The three patients undergoing open surgery had clear cell renal carcinomas. One patient also had MSK, whereas the other two patients lacked stones (as confirmed by histopathological examination of the healthy portion of their kidney) and lacked a family history of stones. The carcinomas were localized within the kidneys, with no evidence of local invasion or metastasis. The patient with MSK was an 80-year-old female found to be heterozygous for the rare -27 + 18G>A variant of the GDNF gene during a case–control association study recently published by our group 9. Other members of the patient's family who carried the same variant also suffered from MSK. The other two patients were females, aged 76 and 56, who were considered to serve as controls because these patients had no MSK or GDNF mutations.

Papillary samples were dissected from the healthy portion of the patients' kidneys immediately after their removal, and the specimens were fixed in 10% formalin in 0.2 M phosphate buffer for light microscope histology or cut into small tissue blocks for cell cultures. The study was conducted according to the principles of the Helsinki Declaration and was approved by the local IRB. All patients provided informed consent according to the indications of the IRBs at the Universities and General Hospitals of Padova and Verona.

### Pathological examination of renal biopsies

For light microscope histology, paraffin-embedded 5-μm-thick sections were stained with haematoxylin and eosin and with PAS. The von Kossa staining method 10 was used to reveal calcium concretions, and scanning electron microscopy (SEM) was used to analyse their composition.

For SEM, ultra-thin sections were cut from Epon-embedded tissue blocks, counterstained with uranyl acetate followed by lead citrate, and examined with a Hitachi H-700 electron microscope. (Hitachi, Chiyoda, TKY)

### Cell cultures

First, papillary tissues were cut into small pieces and incubated in PBS containing 2 mg/ml collagenase I (Sigma-Aldrich Saint Louis, MO, USA) at 37°C for 1 hr, centrifuged (500 × g for 3 min.), and then resuspended in standard culture medium (DMEM-F12; Celbio Milano, Italy). Cells were grown on plastic without any coating and incubated in a humidified atmosphere (5% CO_2_, 37°C). When nearly confluent, the cells were sub-cultured in a 1:1 or 1:2 split ratio using trypsin EDTA (Euroclone Milano, Italy). The cells were cultured up to the 5th passage (p5). At each passage, the cells were seeded in 25-mm^2^ plastic flasks for mRNA extraction and on eight-chamber plastic slides for immunocytochemistry, histochemical staining and SEM.

The control cells were seeded on six-well plastic plates and incubated with standard medium supplemented with 10 mM β-glycerophosphate disodium salt, 50 μg/ml ascorbic acid and 10^−8^ M dexamethasone to trigger mineralization. Different induction protocols were used, including pre-treatment for 48 hrs with dexamethasone and subsequent incubation with β-glycerophosphate disodium salt and ascorbic acid according to Kumar *et al*. 7, incubation with β-glycerophosphate disodium salt and ascorbic acid alone, or incubation with a medium containing dexamethasone, β-glycerophosphate disodium salt and ascorbic acid. Calcification media were replaced every 2 or 3 days for 10–15 days. Commercially available osteogenic medium (Miltenyi Biotec Bologna, Italy) was also used according to the manufacturer's instructions.

### Cell immunophenotyping

Immunocytochemistry was used to assess the presence of von Willebrand factor, vimentin, cytokeratin 8/18, ZO1, CD133, αSMA, desmin and PAX2. The following monoclonal and polyclonal antibodies were used: ZO1 (Santa Cruz Biotechnology Santa Cruz, CA, USA), von Willebrand factor (Dako Carpinteria, CA, USA), vimentin (Dako), cytokeratin 8/18 (Koma Biotech Seoul, Korea), CD133 (Miltenyi Biotec), αSMA (Chemicon Billerica, MA, USA), Desmin (Medac GmbH WEDEL, Germany) and Pax2 (Zymed USA).

The cells on the eight-chamber plastic slides were fixed in ice-cold acetone for 5 sec. and stored at −20°C, and the immunocytochemical analysis was conducted as previously described 11. Slides treated with polyclonal rabbit antibodies were incubated with HRP-labelled anti-rabbit polymer (Envision system; Dako).

At p4, the MSK cells were analysed for surface antigen expression by direct flow cytometry (FACSCalibur, Becton Dickinson New Jersy, USA), as described in detail elsewhere 12, using fluorescein isothiocyanate-conjugated anti-CD34, anti-CD117, anti-intracytoplasmic cytokeratin, and anti-HLA-DR antibodies.

Both the control mesenchymal cells and the MSK cells were analysed at p4 to determine the presence of the CD-146 antigen using MACS Technology (Miltenyi Biotec) with anti-CD146 microbeads according to the manufacturer's instructions.

### Mineralization studies

The cells were examined for biomineralization phenomena. Histochemical staining for alkaline phosphatase (ALP) was performed with a commercial kit (Sigma-Aldrich), calcium crystal staining was performed with von Kossa reagent, and SEM analysis was performed directly on cells grown on plastic slides according to the protocol described in Conconi *et al*. 13.

Immunocytochemistry was used to identify an osteoblast-like phenotype. The cells were stained with monoclonal and polyclonal antibodies against osteocalcin (QED Bioscience San Diego, CA, USA), osteonectin (Chemicon), osteopontin (Chemicon) and Runx2 (Abnova Taipei, Taiwan), and the slides were processed using the same procedure described for immunophenotyping.

### mRNA profiling

Total RNA was extracted from cell cultures at passages 1, 2 and 4 using an RNeasy® Micro Kit (Qiagen Limburgo, NL) according to the manufacturer's instructions. RNA quality and quantity were assessed by spectrophotometric analysis using NanoDrop ND-1000 (Thermo Scientific Waltham, MA, USA) and by capillary electrophoresis using Agilent 2100 Bioanalyzer (Agilent Technologies Santa Clara, CA, USA). A quantity of 70 ng of total RNA was retrotranscribed in a final volume of 20 μl containing 5 mM MgCl_2_, 1 mM dNTPs, 2.5 μM random hexamers (Applied Biosystems), 1 U/μl RNAse Inhibitor (Applied Biosystems) and 2.5 U/μl MuLV reverse transcriptase (Applied Biosystems) in a buffer of 50 mM KCl and 10 mM Tris-HCl (pH 8.3). The reaction was performed at 42°C for 30 min. and at 99°C for 5 min. Three different RT reactions were prepared from each RNA sample.

Real-time RT-PCR was used to quantify osteonectin and osteopontin. Two different housekeeping genes, G3PDH and 18S, were assessed as reference genes (Table1). An iCycler Thermal Cycler (Bio-Rad Hercules,CA,USA) and SYBR Green I technology were used as reported elsewhere 11. PCR standards for each target and housekeeping genes were prepared and amplification efficiency and specificity of each gene were also assessed as previously reported 11. The quantification data were analysed using iCycler software (Bio-Rad) and expressed as the ratio of the starting quantity mean of the target to the housekeeping gene.

**Table 1:** Primer sequences

Gene	Upstream primer	Downstream primer	Size (bp)	TM (°C)	Cycle
Quantitative comparative PCR					
GDNF	ggctatgaaacccaaggaggaactg	tgcctgccctactttgtcactc	135	68	36
Oct4	gacaacaatgaaaatcttcaggaga	ttctggcgccggttacagaacca	218	60	34
Nestin	cagcgttggaacagaggttgg	tggcacaggtgtctcaagggtag	346	60	32
Runx-2	cctctgacttctgcctctgg	tatggagtgctgctggtctg	295	60	40
18S	gtaacccgttgaaccccatt	ccatccaatcggtagtagtagcg	156	60	19
G3PDH	tccaccaccctgttgctgta	accacagtccattgccatcac	450	60	30
Real-time PCR					
GDNF	ggctatgaaacccaaggaggaactg	tgcctgccctactttgtcactc	135	68	
Osteonectin	cctggatcttcttctccttgc	atcaggcagggcttcttgct	71	60	
Osteopontin	cgagacctgacatccagtacc	gatggccttgtatgcaccattc	94	62	
18S	gtaacccgttgaaccccatt	ccatccaatcggtagtagtagcg	156	60	
G3PDH	gaaggtgaaggtcggagt	tggcaacaatatccactttacca	92	62	

Quantitative comparative RT-PCR was used to study GDNF, Oct4, nestin and Runx2 gene expression and 18S and G3PDH mRNA were also assessed as internal standards (Table1). To obtain quantitative data, the kinetic strategy was applied to determine the appropriate number of cycles for quantifying PCR products, as previously described 11,14 (Table1). The Agilent 2100 Bioanalyzer was used to visualize and quantify the PCR products. The relative gene expression levels of GDNF, Oct4, nestin and Runx2 were calculated as the ratio between the quantity (ng/μl) of the target gene PCR product and that of the housekeeping genes. The final quantitative data were normalized to the expression level of the control epithelial cells at p2.

### GDNF knockdown in the HK-2 cell line

The HK2 immortalized proximal tubule epithelial cell line was used for these experiments. The cells were grown in DMEM-F12 (Euroclone) and maintained at 37°C with 5% CO_2_. To obtain stable GDNF-silenced cells, five different shRNAs (Sigma-Aldrich) targeting human GDNF (NM-000514) were used. Each plasmid was transfected separately and a sixth transfection was performed with all five plasmids together according to the manufacturer's protocol (Mirus Madison, WI, USA). The empty shRNA pRS plasmid was used as a negative control. The transfected cells underwent several weeks of selection with 0.75 μg/ml puromycin (Sigma-Aldrich), and different resistant clones were obtained from each 29mer shRNA targeted against GDNF mRNA and the corresponding negative controls. GDNF mRNA expression was evaluated in all clones by real-time RT-PCR using the comparative Ct method (ΔΔCt), and using G3PDH and 18S as reference genes. Relative quantification was performed by determining 2^−ΔΔCt^. Among the silenced clones, the two that showed the greatest level of silencing were chosen for subsequent experiments. GDNF silencing was also assessed at the protein level in these clones by immunocytochemistry using polyclonal GDNF antibody (Santa Cruz Biotechnology).

Two clones in which HK2 was efficiently silenced (shRNA 3D and shRNA 2B) and one negative control clone were cultured in six-well plates at a density of 3 × 10^5^ cells per well in commercially available osteogenic medium (Miltenyi Biotec) for 15 days according to the manufacturer's instructions. Control conditions were established by culturing cells in DMEM-F12. The osteogenic medium was replaced every 2 or 3 days for up to 15 days, and von Kossa staining and SEM were used to detect and examine crystal deposition. Real-time RT-PCR was performed to measure osteopontin and osteonectin expression. The ratio of osteonectin to osteopontin mRNA levels was calculated and graphed. Osteogenic stimulation experiments were replicated three times for the 3D clone and twice for the 2B clone.

### Statistical analysis

The data are expressed as the means ± SE. Statistical analysis was performed with the *t*-test. A *P* < 0.05 was considered statistically significant.

## Results

### Histopathological findings in papillary tissue from an MSK patient

We discovered a renal mass diagnosed as a renal carcinoma in an 80-year-old female with MSK who was heterozygous for the rare variant c.-27 + 18G>A in the 5 UTR region of the GDNF gene, which encodes glial cell-derived neurotrophic factor 9. She underwent open surgery for nephrectomy. On visual inspection, papillary tissue was dissected from the portion of the kidney far from the tumour site in both MSK and control patients, and specimens were handled for histology or cut into small tissue blocks for cell culture. Haematoxylin and eosin staining confirmed that the specimens were papillary tissue.

**Fig 1:**
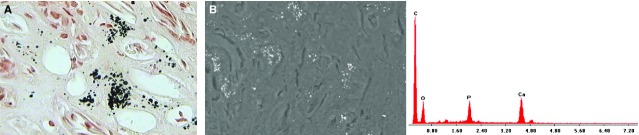
Histopathological findings in papillary tissue from the MSK patient. (A) Light microscopy image (400×) of a von Kossa-stained section. Calcium is visible in the papillary interstitium as round black deposits. (B) A SEM image and spectrum confirm the presence of Ca_2_PO_4_.

**Fig 2:**
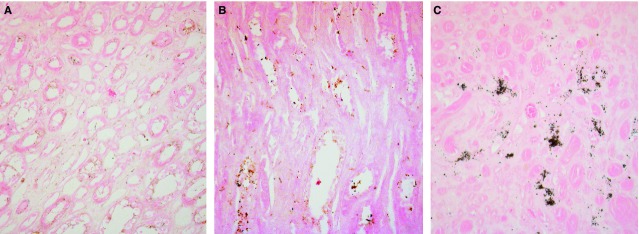
von Kossa staining of control papillary tissues. Light microscopy images (200×) of von Kossa-stained sections of control (A and B) and MSK (C) renal papillary interstitium. No calcium is visible in control tissues.

Von Kossa staining of the MSK specimen identified granular mineral deposits forming clusters in the interstitium, which were between and extremely close to the loops of Henle (Fig.1A). The tubulo-interstitium appeared normal, with no infiltrate or scarring. No intratubular nephrocalcinosis was apparent. The ESEM analysis revealed that the round granular concretions, ranging from 0.7 to 3.5 μm, contained abundant calcium and phosphate (Fig.1B). The control tissues were normal, and no nephrocalcinosis was observed (Fig.2A and B).

### Formation of calcifying nodules by papillary MSK cells

**Fig 3:**
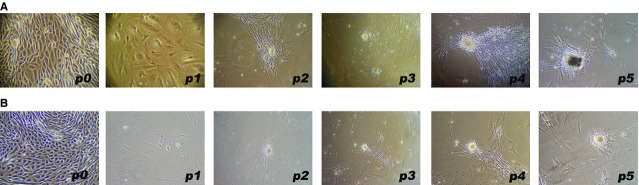
Primary cell cultures from papillary tissues. Phase contrast light microscopy images (100×) of the growth behaviour of MSK cells (A) and mesenchymal control cells (B). Note the close similarity between the two types of cells, except for the presence of dark spots in the MSK nodule at p5.

**Fig 4:**
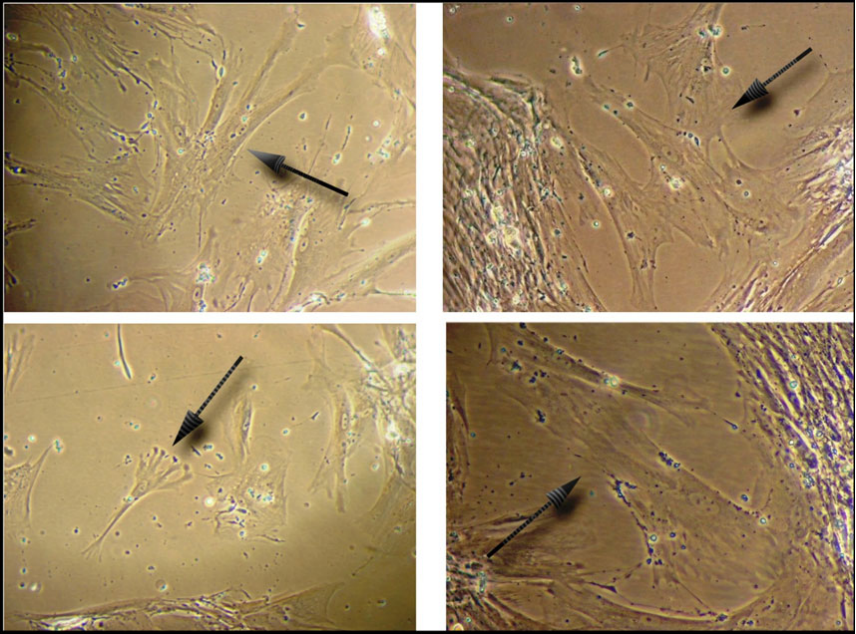
Pericyte-like morphology of MSK cells. p2 MSK cells displaying a mesenchymal morphology (200×); arrows indicate a pericyte-like or mesenchymal stromal cell-like morphology.

After collagenase digestion of the MSK specimen, the cells that were collected and seeded on a plastic surface exhibited a heterogeneous morphology and immunophenotype at p1 (Fig.3A). These cells were maintained in standard culture medium for up to 5 months. Growth was quite slow, and the cells displayed homogeneous growth and morphology from p2 onwards (after 1 month). These cells appeared elongated and flattened or similar to stellate cells with ruffled edges, often with long filamentous processes apparently joining adjacent cells (Fig.4), suggestive of the morphology of pericytes or mesenchymal stromal cells. Interestingly, at confluence, the cells exhibited a spontaneous tendency to grow focally in multiple layers, acquiring a nodular organization (Fig.3A). The formation of nodules began with a break in cell contact at the periphery of a multilayered area, resulting in retraction, with the entire area pulling into a small aggregate or nodule.

**Fig 5:**
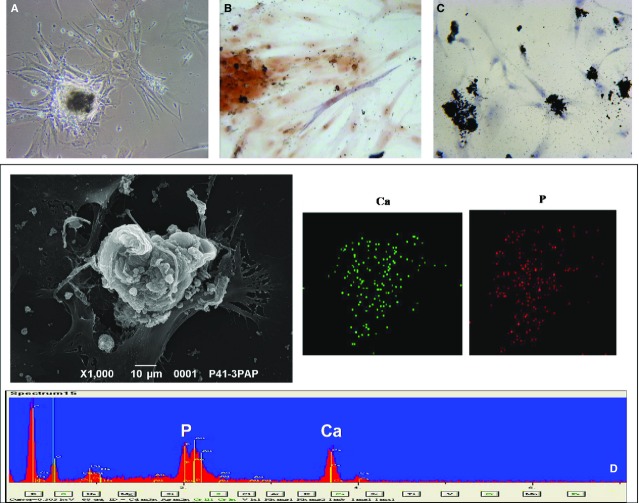
Detection of Ca_2_PO_4_ in MSK nodules. (A) Phase contrast light microscopy image (200×) of a nodule showing dense deposits. (B) Light microscopy image (400×) of cells positive for alkaline phosphatase around the nodules. (C) Light microscopy image (200×) of von Kossa staining reveals calcium deposits in some cells and nodules. (D) SEM images and spectrum confirm that calcifications in the nodules consist of Ca_2_PO_4_ and that calcium (green dots) and phosphorus (red dots) colocalize. The figures are representative of the results of three experiments.

At p4-p5 (after 4–5 months), the nodules became mineralized; this mineralization was visible using light microscopy (Fig.5A), von Kossa staining and SEM analysis (Fig.5C and D). Calcium and phosphate were abundant and colocalized, and some cells adjacent to the nodules were positive for ALP (Fig.5B).

This spontaneous mineralization phenomenon was not observed in the control cultures. The cells in the papillary cell culture from one of the controls showed a typical epithelial morphology and immunophenotype and grew out for three passages (until p3) without showing alterations in morphology (epithelial control cells). The other control cells displayed a mesenchymal morphology and a growth behaviour extremely similar to that of the MSK cells (mesenchymal control cells); their growth was slow, and nodules began to appear spontaneously after 1–2 months, starting from p2 and continuing until p5 (after 8–9 months) (Fig.3B). However, these nodules did not spontaneously become calcified.

### Characterization of calcifying MSK cells

**Fig 6:**
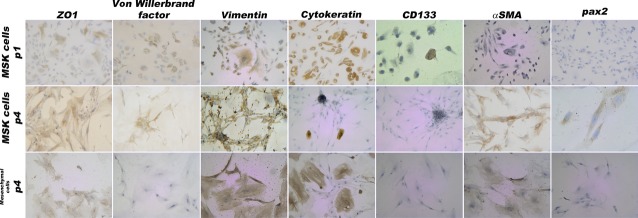
Immunophenotype of MSK and control mesenchymal cells. Light microscopy images (100× and 200×) of MSK cells at p1 and p4 and of control cells at p4; at these time-points, the two types of cells were extremely similar. In total, 100% were positive for vimentin and ZO1, 80% were positive for αSMA and 100% were negative for CD133. At p1, 90% of the MSK cells were positive for cytokeratin and vimentin; 10% were positive for von Willebrand factor; and 40% were positive for ZO1, thus indicating phenotypic heterogeneity. The figures and percentages are representative of the results of three experiments.

We attempted to identify the phenotype of the calcifying cells by immunocytochemistry, FACS and MACS analyses. At p4, the MSK cells were CD34^−^, CD177^−^, HL-DR–, CD133^−^, E-Cadherin– and desmin–; a subpopulation of the cells was CD146^+^. The MSK cells were positive for vimentin, ZO-1, and alpha SMA; a subpopulation of flattened cells also stained positive for cytokeratin, and a few cells stained positive for von Willebrand factor. Evidence of PAX2 positivity was observed; however, this positivity was unexpectedly cytoplasmic (Fig.6).

**Fig 7:**
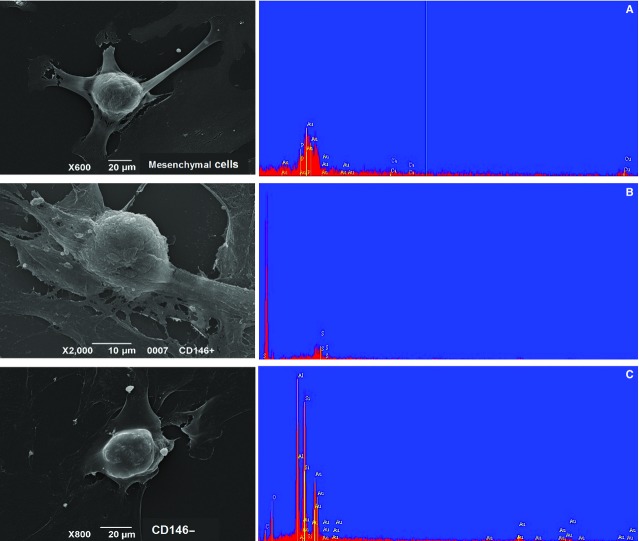
SEM analysis of control cells with a growth behaviour identical to MSK cells. SEM images and spectra of (A) mesenchymal control cells, (B) CD146-positive and (C) CD146-negative mesenchymal control cells. The cells were incubated for 15 days with commercial osteogenic medium purchased from Miltenyi Biotec. The spectra revealed that calcium and phosphorus were absent.

Immunophenotyping of the mesenchymal control cells showed that these cells were positive for vimentin, ZO-1 and alpha SMA, and a subpopulation of flattened cells stained positive for cytokeratin (Fig.6). Therefore, this phenotype was extremely similar to that of the MSK cells, except that this phenotype lacked any PAX2 or von Willebrand positivity. Using magnetic beads coated with CD146 mAb, mesenchymal control CD146^+^ and CD146^−^ cells were isolated and incubated with inducing media; however, no nodular calcification was apparent (Fig.7).

**Fig 8:**
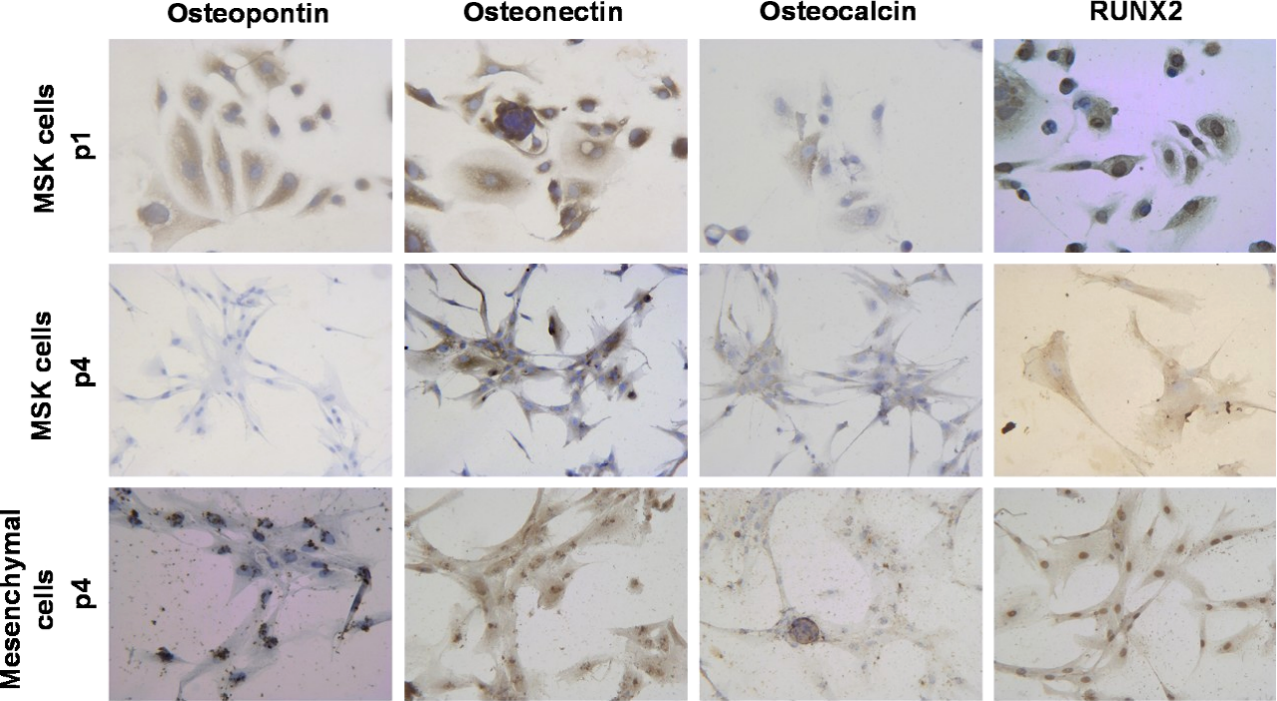
Immunocytochemistry for osteoblastic markers of MSK and control mesenchymal cells. Light microscopy images (200×) of MSK cells at p1 and p4 and of mesenchymal control cells at p4, showing 100% positivity for osteonectin; 40% of the cells were faintly positive for osteocalcin. Osteopontin was expressed by 100% of MSK cells at p1 and by 60% of mesenchymal cells (localized around the nuclei). Note the different localization of Runx2 immunopositivity, which was nuclear in MSK cells at p1 and in mesenchymal cells at p4, whereas Runx2 immunopositivity was surprisingly cytoplasmic in MSK cells at p4. The figures and percentages are representative of the results of three experiments.

Subsequently, we searched for osteogenic markers. Upon immunocytochemical examination at p4, MSK cells and nodules were positive for osteonectin and Runt-related transcription factor 2 (Runx2), weakly positive for osteocalcin, and negative for osteopontin (Fig.8). Some cells near the nodules were positive for ALP (Fig.5B) as well as for collagen I and laminin, which are typical bone extracellular matrix proteins (data not shown). By comparing the MSK cell phenotype at p4 with that at p1, p1 cells clearly displayed the same osteogenic profile, except for two striking differences: the presence of osteopontin and the nuclear localization of Runx2 (Fig.8).

To understand why the mesenchymal control cells did not form mineralized nodules, we compared their osteogenic profile at p4 with that of the MSK cells and found that the mesenchymal control cells resembled MSK cells at p1. Remarkably, the mesenchymal control cells expressed osteopontin and stained positive for nuclear Runx2 (Fig.8).

### Osteogenic mRNA profile of MSK cells

The selection of appropriate reference genes for data normalization in gene expression analysis is one of the essential steps in the experimental design 15. We used two different housekeeping genes, G3PDH and 18S – one of medium abundance and the other of high abundance respectively – and validated their use as reference genes in our experimental setting. By real-time RT-PCR, we compared the expression levels of the two housekeeping genes in the different experimental conditions to be tested. Applying regression analysis, strict and significant correlations were found between osteonectin/G3PDH and osteonectin/18S ratios (*r* = 0.80, *P* = 0.003) and between osteopontin/G3PDH and osteopontin/18S ratios (*r* = 0.94, *P* = 0.000). Relying on these results, we adopted 18S as reference gene. We were aware that the use of multiple reference genes is the optimal choice for accurate RT/PCR expression profiling 16, however the low yield of total RNA obtained from MSK and mesenchymal primary cells did not allow us to apply this more accurate normalization.

**Fig 9:**
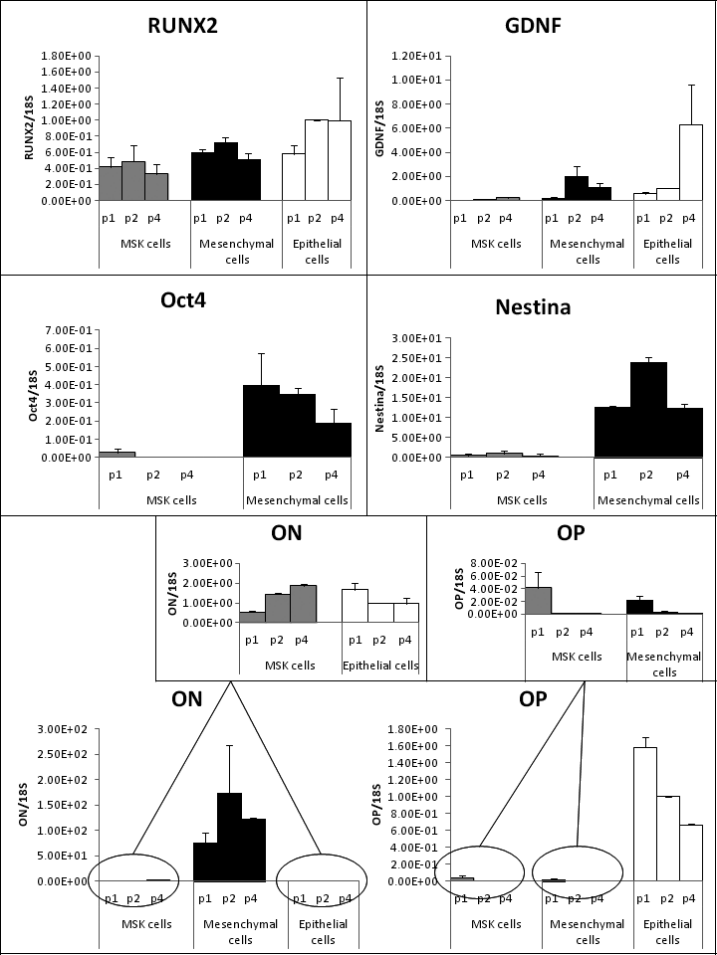
mRNA profiles of MSK and control cells. The mRNA levels of six different genes at different passages (p1, p2 and p4) detected by quantitative comparative RT-PCR (Runx2, GDNF, OCT4 and Nestin) and by real-time RT-PCR (ON: osteonectin; OP: osteopontin) are shown as ratios of the target genes to reference gene and normalized to p2 control epithelial cells. The results represent the mean ± SD of three separate RT/PCR experiments.

An exploration of the differences between the osteogenic profiles of MSK and control cells at the mRNA level revealed that Runx2 and osteopontin were down-regulated in both MSK and control mesenchymal cells compared with control epithelial cells. This finding confirmed the marked similarity of the two types of primary papillary cells and demonstrated the opposite trend in osteonectin expression in MSK *versus* epithelial control cells (*i.e*. osteonectin was up-regulated in MSK cells during the various passages but was down-regulated in epithelial cells). However, a striking difference was noted in the osteonectin mRNA level, which was highest in the mesenchymal control cells (Fig.9). The divergent expression of osteopontin *versus* osteonectin in the MSK cells is also notable because osteonectin is known to trigger mineral deposits 17, whereas osteopontin is considered a powerful inhibitor of crystal formation 18–20.

### GDNF expression in MSK cells

We explored the possible influence of the GDNF mutation carried by MSK cells on the level of GDNF expression. Using quantitative comparative RT-PCR, relative GDNF mRNA levels were examined in MSK *versus* control cells. The results demonstrate that the MSK cells contained undetectable levels of the GDNF transcript at p1 and exhibited only weak expression of GDNF at p2 and p4, whereas GDNF mRNA was observed in the control cells beginning in the first passages (Fig.9). Notably, the marked difference in the regulation of GDNF expression between the MSK and control cells was evident at every culture passage.

### GDNF knockdown in the HK-2 cell line

**Fig 10:**
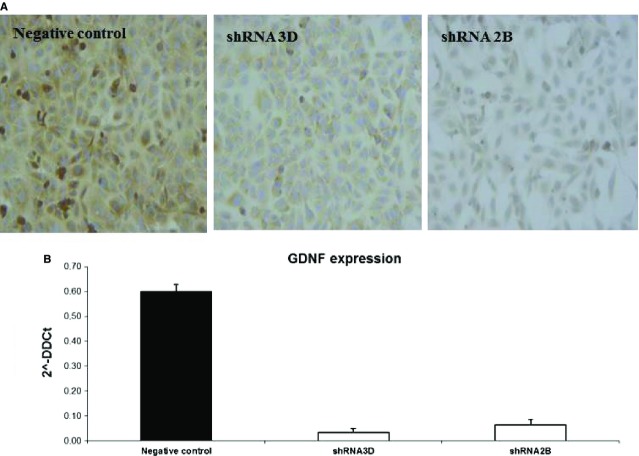
GDNF knock-down in the HK2 cell line. (A) Detection of the GDNF protein in silenced clones. GDNF immunostaining with a monoclonal anti-GDNF antibody revealed a reduction in signal between silenced (shRNA 3D, shRNA 2B) and negative control clones. The figures are representative of the results of three experiments. (B) Real-time PCR quantification of GDNF mRNA expression in silenced (white bars) and control clones (black bar). The results represent the mean ± SD of three separate experiments performed in triplicate.

**Fig 11:**
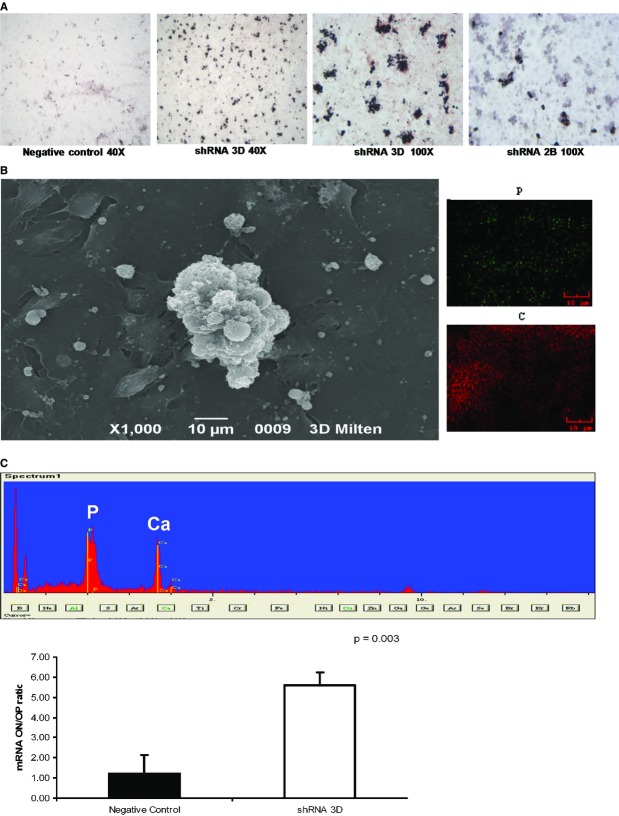
Detection of Ca_2_PO_4_ deposits in HK2 cells cultured in osteogenic medium. (A) Light microscopy images (40× and 100×) of von Kossa staining reveal calcium deposits in cell aggregates. The low magnification image reveals that the dark deposits were much more abundant in the silenced clones than in the control clones. The figures are representative of the results of three experiments. (B) SEM images and spectrum confirming Ca_2_PO_4_ deposition. Calcium (green dots) and phosphorus (red dots) were colocalized. (C) Real-time PCR results for the ratios of osteonectin to osteopontin mRNAs, indicating that the balance between pro- and anti-osteogenic factors in the silenced clones was tipped in favour of an osteogenic process. The results represent the mean ± SD of three separate experiments performed in triplicate.

To determine whether GDNF down-regulation could trigger or promote the calcification process in renal cells, we performed a stable shRNA knockdown of GDNF in HK2 cells. In two clones, the gene knockdown resulted in an approximate 90% decrease in GDNF transcript levels with a less marked reduction in protein levels (Fig.10). Culturing the silenced cells in osteogenic medium for 15 days led to the deposition of Ca_2_PO_4_ aggregates, as revealed by von Kossa staining and SEM (Fig.11A and B). However, a considerably smaller number of these aggregates were observed in the negative control (Fig.11A). No Ca_2_PO_4_ deposits were found in the negative control or in silenced clones cultured under normal conditions. To determine whether this calcium deposition was related to an osteogenic-like process, experiments were performed with real-time RT-PCR to measure osteopontin and osteonectin expression. At 15 days, when the Ca_2_PO_4_ deposits were clearly evident, the osteonectin/osteopontin ratio was significantly higher in the silenced HK2 cells compared with the control cells (Fig.11C).

## Discussion

The most accredited hypothesis that can explain the formation of interstitial nephrocalcinosis is that spontaneous calcium-phosphate crystallization occurs in the interstitium because this region is oversaturated with calcium-phosphate salts 2,3. Currently, the exact role of the interstitial cells in response to the arrival of these potentially precipitating ions is unknown.

Here, we demonstrate for the first time that papillary renal cells are capable of spontaneously forming Ca_2_PO_4_ nodules *in vitro*.

To investigate whether the spontaneous mineralization observed in the MSK patient's cells was merely a physicochemical phenomenon or a highly organized biomineralization process, we searched for any signs that bone mineralization machinery was being expressed in the cells. We found that the cells were positive for osteogenic markers, such as osteonectin, ALP, collagen I, laminin and Runx2, and weakly positive for osteocalcin but negative for osteopontin, which is a known inhibitor of crystal formation. The up-regulation of osteonectin and the down-regulation of osteopontin were also demonstrated at the mRNA level. However, mesenchymal control cells exhibited a very similar pattern, except for two striking differences: the localization pattern of Runx2, which was nuclear in control cells and cytoplasmic in MSK cells, and the immunopositivity for osteopontin. Runx2 is considered the master transcriptional regulator of osteoblastogenesis, and it exerts its function early during the osteogenic process by controlling the differentiation of mesenchymal precursors into pre-osteoblasts 17,21,22. Our results prompted us to speculate that the osteogenic process observed in the MSK cells at p4 was more advanced because the cells were already osteoblast-like, whereas the control mesenchymal cells remained in an earlier stage (*e.g*. that of mesenchymal precursors). The impression that the mesenchymal control cells resembled progenitors also stemmed from the higher levels of nestin and OCT4 in these cells compared with the nestin and OCT4 levels in the MSK cells (Fig.9). On the basis of this hypothesis, we attempted to induce the osteoblastic phenotype in mesenchymal cells by incubating these cells in osteogenic medium for 15 days. However, we did not observe any Ca_2_PO_4_ deposition. Fourteen days of induction may not have been long enough to trigger any osteoblastic differentiation; alternatively, other inductive milieus may be required to modify this type of renal mesenchymal stroma-like cell.

Our results indicate that a biomineralization phenomenon similar to that described for pericytes and for VSMCs in culture occurred in the MSK cells.

Very few cell types (*i.e*. bovine retinal pericytes 23, human and bovine vascular smooth muscle cells 24, and mesenchymal stromal stem cells (MSCs) 25) are capable of growing in such a pattern. Pericytes are relatively uncommitted cells and, together with the MSCs, they form a set of multipotent cells belonging to the perivascular niche 26.

The morphology, immunophenotype and growth behaviour of MSK and mesenchymal control cells suggested that they resembled MSCs, as they were positive for ZO1, alpha SMA, vimentin, and CD146. This result confirmed that the renal papilla might also be a reservoir of stem/progenitor cells in humans 27,28 and supported the hypothesis previously suggested by our group 29 that stem cells in the human renal papilla are most likely associated with the perivascular niche. MSCs may be derived from perivascular cells, and pericytes are good candidates for representing the MSC population in the kidney, similar to other organs 26. The microvasculature of the renal papilla is particularly rich in pericytes, which regulate microvascular integrity in the peritubular capillary network and give the descending vasa recta their contractile function 30. Thus, similar to VSMCs, papillary MSCs linked to the perivascular niche may be capable of driving an osteogenic process under certain conditions.

Regarding the mechanism underlying the spontaneous mineralization process that was observed in the papillary cells from our MSK patient, we surmise that the GDNF mutation could have influenced the particular behaviour of the cultured cells. The rare mutation in the GDNF promoter region carried by the MSK cells could affect GDNF expression. In fact, the GDNF mRNA level was much lower than that in either of the controls, including the mesenchymal cells. Mesenchymal control cells are very similar to MSK cells but express a higher level of GDNF; however, they were less likely to differentiate towards an osteoblastic phenotype compared with MSK cells. In fact, mesenchymal control cells did not form mineralized nodules spontaneously or after induction.

Although we were aware that the difference in GDNF expression might not fully explain the differences in the mineralization behaviour observed *ex-vivo*, we wanted to investigate the hypothesis that GDNF down-regulation might trigger a mineralization process in human renal cells. To accomplish this, we performed stable shRNA knockdown of GDNF in HK2 cells. Under osteogenic stimulation, the silenced cells displayed a greater capacity to synthesize Ca_2_PO_4_ than control cells by regulating the osteonectin/osteopontin ratio in favour of osteonectin. This result demonstrates a role for GDNF in the calcification process of the HK2 cells.

The fact that control cells placed in an osteogenic medium were also capable of undergoing a biomineralization process is noteworthy and confirms the results of Kumar *et al*. 7 in a rat renal cell line. We are aware that HK2 cells are quite different from papillary mesenchymal cells and that the results we obtained cannot completely prove that GDNF was responsible for the abnormal behaviour of the MSK cells. Nevertheless, we believe that our findings might provide insight regarding an unexpected function of GDNF in the adult kidney. GDNF is known to have survival properties in neurons 31. In addition, in the kidney, GDNF was demonstrated to act in an autocrine manner to support survival in podocytes 32. In MSK cells lacking GDNF, recovery from noxious stimuli may not have occurred, leading cells to express cell death programs, thus triggering the calcification process. In fact, we know that vesicles, some of which are derived from apoptotic VSMCs, seem to drive the calcification process during vascular calcification 24. This observation suggests that apoptosis might be a crucial initiating event in the calcification process.

The precise role of GDNF in the adult human kidney must be investigated in greater depth to elucidate the molecular mechanisms by which GDNF loss could favour the cell calcification process.

In conclusion, our results indicate a possible role for resident papillary renal cells, which were identified as mesenchymal or pericyte-like cells, in the pathogenic mechanisms leading to nephrocalcinosis. Under specific pathological conditions (MSK or possibly other metabolic conditions), these cells might be capable of differentiating into osteoblast-like cells and might produce Ca_2_PO_4_ deposits.

Knowing not only the site of initial crystallization but also – more importantly – the mechanisms underlying crystal generation could improve our understanding of stone formation and enable investigators to examine more focused hypotheses, thereby helping researchers to discover therapies for preventing recurrent nephrolithiasis.
